# Effects of a dry-land strengthening exercise program with elastic bands following the Kabat D2 diagonal flexion pattern for the prevention of shoulder injuries in swimmers

**DOI:** 10.3389/fphys.2023.1275285

**Published:** 2023-11-13

**Authors:** Ivan Della Tommasina, Alfonso Trinidad-Morales, Pedro Martínez-Lozano, Ángel González-de-la-Flor, José Ángel Del-Blanco-Muñiz

**Affiliations:** ^1^ Faculty of Physical Activity and Sport Sciences, European University of Madrid, Madrid, Spain; ^2^ Faculty of Education and Humanities, European University of Madrid, Madrid, Spain

**Keywords:** swimming, shoulder injuries, exercise, rotator cuff, elastic band

## Abstract

**Background:** During the repetitive execution of the swimming strokes, the muscles responsible for the internal rotations of the shoulders tend to become stronger compared to the muscles that oppose these movements. The aim of this study was to analyse the effect of a strengthening program for the shoulder rotator muscles using elastic band exercises in a diagonal Kabat pattern (D2 for flexion) in swimmers, to develop an effective, quick and easy-to-implement protocol for preventive training routines.

**Methods:** A randomized controlled trial design was carried out. Internal and external rotation range of movement, isometric strength of the muscles responsible for internal and external rotation of the shoulder, scapular movements, was measured at the beginning of the study and after 8 weeks post-intervention. A total of 22 male swimmers participated in the study and were randomly assigned to either an experimental group (*n* = 11) or a control group (*n* = 11). The experimental group underwent a 8-week shoulder-strength program using elastic bands, while the control group focused on aquatic training.

**Results:** The strength-training program resulted in an improvement in the isometric strength of the muscles responsible for external rotation and a better balance between the shoulder rotator muscles in the experimental group. However, these improvements have not been significant (*p* > 0.05).

**Conclusion:** The strengthening exercise program showed minimal improvement in shoulder rotation strength and range of motion. These findings suggest that the prescribed shoulder-strengthening exercise could be a quick-beneficial dry-land training option to improve external rotation shoulder strength or range of motion, but more studies with larger sample sizes and more weeks of treatment are needed to determine the efficacy of this protocol.

## 1 Introduction

Swimming is a cyclical sport with high technical demands, training loads and strength requirements to overcome an external load on the upper body (Jürimäe et al., 2007). The leverage effect of the upper limbs in the water can lead to joint and muscle overload and even injury (Hill et al., 2015). The rotator cuff in particular plays an important role in this process, as it is involved in stabilizing the shoulder against the addiction and internal rotation movements required to propel the swimmer through the water (Batalha et al., 2015). However, during the training season, overuse of the internal rotators and shoulder adductors can lead to a strength imbalance in swimmers, as the internal rotators gain much more strength compared to the external rotators and shoulder abductors, weakening the latter by the end of the season (Trinidad et al., 2021). This reason, an imbalance between these antagonistic muscle groups can lead to rotator cuff injuries known as “swimmer’s shoulder” (Batalha et al., 2015; Kluemper et al., 2016). As a preventive measure, strength and stretching exercises have been incorporated into training in various sports disciplines to improve the performance of athletes (Asker et al., 2018). It would thus be advisable to develop protocols to prevent shoulder injuries caused by these muscular imbalances by strengthening the muscles responsible for external rotation and abduction of the shoulder (Marek et al., 2014).

In terms of etiology, prevalence and incidence, the shoulder is the joint with the greatest range of motion in the whole body, so the incidence of injury is very high (Tooth et al., 2020). In so-called “overhead athletes,” which include swimmers, injuries to this joint complex have a high prevalence (Gaunt and Mafulli, 2012; Wanivenhaus et al., 2012). However, the incidence of shoulder pain in swimmers is around 38%, with between 29% and 91% of swimmers having experienced this symptom during their sporting career (Bak, 2010). This could be explained by the fact that in water, propulsion is based on the upper limbs, in contrast to land sports where the lower limbs are predominantly used (Sein et al., 2010). In addition, overuse of the shoulder muscles during weekly training sessions, which can be as many as 6 or 7 per week, leads to muscle imbalances between the rotators which, if not treated or prevented, can lead to injury later (Tessaro et al., 2017). Furthermore, in terms of therapeutic treatment, the most commonly used techniques to increase rotator cuff strength and muscular balance in overhead athletes include scapular-humeral stabilisation exercises, plyometrics, maximal strength exercises and the use of electrotherapy (Feijen et al., 2020). However, in recent years some studies have been published demonstrating the efficacy of strength exercises with elastic bands following a PNF-type diagonal Kabat pattern (Page et al., 1993; Richards and Dawson, 2009), showing positive effects on the improvement of rotator cuff strength (Moeller et al., 2014) after combining compensatory training with elastic bands with the diagonal Kabat pattern. However, most of these studies analysed sports with similar biomechanics to swimmers, such as handball, volleyball and baseball (Swanik et al., 2002).

In relation to elastic band training, scientific evidence has demonstrated the effectiveness of Kabat diagonal training using elastic bands following a progressive strength training protocol (Page et al., 1993). The elastic bands used in the current studies are characterised by different resistances, indicated by different colours, and are thought to provide greater electromyographic activation of the scapular musculature compared to free weight training (Witt et al., 2011). The protocol proposed by Manske et al. (Manske et al., 2015) shows the progression of the resistance of the elastic band as a function of the subject’s perceived exertion (RPE) after performing the training tasks, using a numerical scale from 0 to 10 (Wong-Baker scale): if the RPE value was lower than 6, the resistance was increased. During the repetitions, each patient’s subjective perception of exertion (RPE) was assessed. If the RPE was equal to or less than 6, the repetitions were increased to a maximum of 25, then the resistance was increased by changing the elastic band and returning to 15 repetitions (Manske et al., 2015). But the position of the patient to perform the Kabat diagonals varies according to the studies, the most common position being seated to avoid thoracic compensations (Escamilla et al., 2009). As for the point of fixation of the elastic band, this varies according to the studies in 3 points: head, feet, and iliac crest. However, there is no single consensus in the current literature on protocols applied to swimmers, with most studies focusing on overhead athletes in water polo, volleyball, baseball and softball (Escamilla et al., 2009; Witt et al., 2011; Manske et al., 2015; Batalha et al., 2018; Park and Park, 2019).

Therefore, training the external rotators of the shoulder may be a strategy to prevent rotator cuff injuries in swimmers due to the high incidence of injuries and the biomechanical importance of the rotator cuff during the sporting gesture [Batalha et al., 2015; Costa et al., 2014). For this reason, the aim of the study was to examine the effects of a diagonal Kabat pattern (D2 for flexion) on the strength of all the movements involving external rotation of the shoulder, scapular movements, and shoulder range of motion (ROM). We hypothesized that the experimental group had an improvement in shoulder strength, ROM and scapular movements.

## 2 Material and methods

### 2.1 Study design

A prospective, longitudinal, randomised, controlled, single-blind, 8-week clinical trial was conducted following the CONSORT guidelines and registered at www.clinicaltrials.gov (No. NCT05884996). Subjects who agreed to take part in the study were randomised into two groups using opaque envelopes: Control Group and Intervention Group (shoulder external rotator cuff strengthening using Kabat diagonal of D2 flexion and progressive resistance using elastic bands). All pre- and post-intervention assessments were performed by 2 assessors blinded to the subject’s group.

### 2.2 Participants

A total of twenty-two university swimmers were included in this study. Inclusion criteria were to be between 18 and 33 years old, male, and to train at least 2 days per week; exclusion criteria were to have no acute infections or to have any shoulder pathology. On the other hand, subjects who did not sign the informed consent form were not included in the study, which was approved by the Research Ethics Committee of the European University of Madrid (code CIPI/20/007) in accordance with the Declaration of Helsinki (World Medical Association, 2001).

### 2.3 Instruments

#### 2.3.1 Maximum isometric strength of the external and internal rotators of the shoulder joint complex

An Active Force 2 dynamometer (San Diego, United States of America) was used according to the protocol of Hébert et al. (2011). Prior to the start of the test, the swimmers were instructed in the entire procedure, given time to familiarise themselves with the equipment and given 3 min to warm up with 2 test sets at minimum effort. The unit of measurement of the device was the kilogram (kg).

Subjects were then placed in the supine decubitus position. The upper limb to be assessed was placed in shoulder abduction and 90° elbow flexion so that the forearm was perpendicular to the ground and the stretcher. The dynamometer was placed transversely to the distal extremity of the upper limb, in precise contact with the patient’s wrist. Two maximal isometric contractions were performed for 5 s, with 30 s rest between contractions, for the external and internal rotation movements. During execution, the blinded physiotherapist maintained firm contact with the dynamometer and applied a parallel resistance equal to the force applied by the athlete, without allowing the swimmer’s upper limb to move. In addition, the athletes were verbally stimulated to produce the maximum possible effort and were instructed not to perform the Valsava manoeuvre during the test. Finally, the force exerted was digitally recorded by the apparatus and only the maximum value achieved was used, as a higher value indicated greater muscular effort.

#### 2.3.2 Range of motion in external rotation

A goniometer (Tandou_1AA800252^®^) was used to measure the swimmers in the supine position on a stretcher, with the arm stabilised and a towel placed under the humerus to maintain a 10/15° arm position anterior to the coronal plane. The athlete’s arms were placed at 90° shoulder abduction, the elbow off the stretcher at 90° flexion, and the forearm and wrist at 0° (perpendicular to the stretcher and floor). The axis of the goniometer was placed over the central part of the acromion. The fixed arm of the goniometer was positioned perpendicular to the floor, while the movable arm was superimposed on the fixed arm, aligned with the longitudinal midline between the ulna and its styloid process.

#### 2.3.3 Muscular balance of the rotator cuff

With the results of the maximum isometric strength assessment, a ratio was made between the internal and external rotators of each arm (maximum isometric strength - maximum internal rotation/maximum isometric strength—maximum external rotation); a value closer to 1 indicates a better muscular balance.

#### 2.3.4 Scapulohumeral coordination using the PALM

The PALM was used to measure the medial/lateral displacement of the shoulder blade and its upward rotation during arm raising and at rest. The swimmers were placed in a standing position and the arms of the PALM (da Costa et al., 2010) were placed corresponding to the inferior angle of the scapula and the spinous process of the closest thoracic vertebra. The distance between the two points was measured at rest and at the end of arm elevation. The results were then compared with the contralateral side.

### 2.4 Procedure

Elastic bands were used during the test, according to the protocol described by Batalha et al. (Batalha et al., 2015) with the aim of increasing the muscle strength of the external rotators and achieving the optimal angle of force application of the external rotators. Each session with the experimental group lasted approximately 4 min per subject and took place 2 days per week during the 8-week period. The intervention took place before the usual aquatic exercise session. The control group had to follow their usual routine without repeating the exercises suggested to the experimental group. The sessions were led by the physiotherapist-researchers of the study, who were responsible for supervising the correct performance of the exercises.

During each repetition, the subjects had to carry the end of the elastic band following Kabat’s D2 diagonal pattern for flexion. All started from a position of adduction, extension and internal rotation of the shoulder with extension and pronation of the elbow, flexion and ulnar tilt of the wrist, to a position of flexion, abduction and external rotation of the shoulder with extension and supination of the elbow, extension and radial tilt of the wrist. Each subject in the experimental group began the first session by performing 3 sets of 10 repetitions for each upper extremity. Each subject in the intervention group performed 3 sets of 10 repetitions for each upper extremity. At the end of each set, each patient’s subjective perception of exertion (RPE) was assessed using a visual scale with values from 0 to 10, depending on the intensity of the perceived exertion. If a value of 6 or less was reached on the RPE, 5 repetitions were added up to a maximum of 20. Once 20 repetitions were reached, the resistance of the elastic band could be increased in the following session, starting again with 10 repetitions.

#### 2.4.1 Sample size calculation

The sample size calculation was performed using the G*Power Software version 3.1.9.2, considering an alpha error of 0.05 and a statistical power of 0.8, with a medium effect size (f = 0.33 or Eta partial squared = 0.10) based on the primary outcome (shoulder strength) and an estimated dropout rate of 10%. Therefore, a total of 22 participants were determined as the required sample size. This sample was divided into two groups, with each group consisting of 11 participants.

### 2.5 Data analysis

Statistical analysis was performed using IBM SPSS Statistics version 29 for Windows (IBM, Armonk, NY, United States). The distribution of the data was assessed using the Shapiro-Wilk test for sample sizes less than 50, in addition to examining histograms. For parametric variables (*p* > 0.05), central tendency and dispersion data were presented as mean and standard deviation, while for non-parametric variables (*p* < 0.05), median and interquartile range were reported.

An independent *t*-test or Mann-Whitney U test was performed to compare baseline characteristics between the two groups, taking into account the assumptions of homoscedasticity and sphericity. If the assumptions were met, a two-way analysis of variance (ANOVA) with a 2 × 2 design was performed. Effect size was assessed using partial eta squared (η2p), with values of 0.01 interpreted as small, 0.06 as medium and 0.14 as large. A 95% confidence interval was used for all analyses.

## 3 Results

A total of 22 participants with a mean age of 25.71 years old, weight of 75.53 kg, height 177.34 cm and 13.32 years of swimming experience were included in the study, divided into two groups: experimental group (n = 11) and control group (n = 11). Table 1 shows the sociodemographic characteristics of the total sample. No differences were observed between the experimental and control group (*p* > 0.05).

**TABLE 1:** Sociodemographic data of the total sample, experimental and control group.

Variables	Total sample (n = 22)	Experimental group (n = 11)	Control group (n = 11)	*p*-value (between-group)
Age (years)	25.71 ± 3.22	25.7 ± 3.4	25.7 ± 2.9	0.981
Height (cm)	177.34 ± 3.52	176.3 ± 2.6	178.5 ± 4.2	0.142
Weight (kg)	75.53 ± 4.31	74.1 ± 3.0	77.1 ± 5.0	0.098
BMI (kg/m2)	23.93 ± 0.64	177.3 ± 3.5	177.3 ± 3.5	0.189
Training frequency (days/week)	2.20 ± 40.44	2.2 ± 0.4	2.2 ± 0.4	0.849
Experience (years)	13.32 ± 6.36	11.8 ± 6.0	15.1 ± 6.5	0.235
Arm Dominance (L/R)	4/18	3/9	1/9	0.594
Swimming breathing side (L/R)	5/17	3/9	2/8	0.999

Abbreviations: BMI, body mass index; L, left side; R, right side. Data are expressed are mean ± standard deviation or frequency

Baseline outcome measures did not show significant differences between groups (*p* > 0.05). There was no group-by-time interaction for the variables related to shoulder range of movement, shoulder muscle strength and scapular position at rest and at maximum flexion (*p* > 0.05) (Table 2). Small to medium effect size was observed in shoulder internal and external rotation ROM, isometric shoulder internal and external rotation strength and scapular position (η2*p* = 0.00—0.10).

**TABLE 2:** Outcome measures at baseline and 8 weeks post-treatment.

Variables	Group	Baseline	Post 8 Weeks		*p*-value time	*η* ^*2*^ *p*
					x group	
Range of Movement (º)						
Right IR	EG	77.09 ± 10.17		80.81 ± 12.21	0.208	0.08
	CG	80.40 ± 7.49		79.18 ± 11.12		
Left IR	EG	80.82 ± 12.21		73.36 ± 12.75	0.263	0.06
	CG	77.90 ± 6.26		76.80 ± 9.98		
Right ER	EG	85.55 ± 12.39		88.91 ± 7.35	0.163	0.10
	CG	91.80 ± 9.08		90.60 ± 5.50		
Left ER	EG	85.27 ± 8.30		88.45 ± 8.85	0.350	0.05
	CG	87.50 ± 8.32		88.70 ± 7.87		
Isometric Strength (kg)						
Right IR	EG	14.92 ± 3.07	14.94 ± 2.51		0.265	0.07
	CG	15.67 ± 3.71	17.71 ± 4.36			
Left IR	EG	16.25 ± 4.76	15.45 ± 1.91		0.447	0.03
	CG	16.16 ± 6.21	16.66 ± 3.68			
Right ER	EG	14.76 ± 2.92	16.49 ± 3.25		0.263	0.10
	CG	13.89 ± 4.35	15.09 ± 3.72			
Left ER	EG	16.04 ± 3.54	16.73 ± 4.47		0.709	0.01
	CG	14.38 ± 4.73	15.73 ± 3.87			
Right ER/IR Ratio	IG	1.06 ± 0.32	0.92 ± 0.15		0.431	0.03
	CG	1.19 ± 0.37	1.19 ± 0.20			
Left ER/IR Ratio	IG	1.02 ± 0.21	0.96 ± 0.21		0.926	0.00
	CG	1.13 ± 0.18	1.08 ± 0.17			
Scapular position (cm)						
Right at Rest	IG	10.27 ± 1.10	10.57 ± 1.65		0.379	0.04
	CG	9.95 ± 1.04	9.88 ± 1.05			
Left at Rest	IG	10.05 ± 1.08	10.18 ± 1.25		0.657	0.01
	CG	9.95 ± 1.01	10.29 ± 1.51			
Right at maximum flexion	IG	16.08 ± 0.87	16.17 ± 0.81		0.233	0.07
	CG	15.53 ± 1.22	15.16 ± 0.67			
Left at maximum flexion	IG	16.13 ± 0.75	15.48 ± 1.44		0.788	0.00
	CG	15.70 ± 0.89	15.21 ± 1.52			

## 4 Discussion

The aim of this study was to analyse the effect of a strengthening programme for the shoulder rotator muscles using elastic band exercises in a diagonal Kabat pattern (D2 for flexion) in swimmers, in order to develop an effective, quick and easy-to-implement protocol for preventive training routines. In contrast with our hypothesis, the participants of the experimental group did not show a significant improvement in shoulder strength, ROM or scapular movement compared to the control group post-intervention.

Training loads generate an imbalance in swimmers between the adductor and internal rotator muscles due to overload, while the antagonist muscles do not receive the same training load. Indeed, there is evidence of a mismatch between the strength developed by these muscle groups, altering the agonist-antagonist relationship (Weldon and Richardson, 2001; Drigny et al., 2020) as a potential cause of injury. However, corrective measures for this imbalance should be accessible to all swimmers, regardless of their competitive level and technical means.

On the other hand, the natural progression that swimmers undergo with training is towards muscle imbalance, according to data observed in young swimmers (15–18 years old), who develop internal rotator strength with training, but lose external rotator strength over 3 years of continuous training (Habechian et al., 2018) It seems logical to assume that the gains in internal rotator strength are part of the expected athletic development with training. Therefore, the loss of strength in the external rotators should alert coaches to take measures to ensure a harmonious development of strength in the shoulder complex. The isometric agonist-antagonist RE/RI strength ratio that would be expected in healthy shoulders should be between 75%–100% (Cools et al., 2016).

However, it is not only muscle strength that is a parameter to be monitored in the prevention of shoulder pathology in swimmers. Limited glenohumeral mobility is also a risk factor (Cejudo et al., 2019). According to a cohort study with a 12-month follow-up (Walker et al., 2012), the risk of shoulder pain is multiplied by 8.1 in swimmers with an excess of range in external rotation and by 12.5 in swimmers with a restriction of joint range in external rotation. However, in a retrospective study, no relationship was found between variability in humeral torsion parameters and rotation ranges with athletes’ history of shoulder injury (Holt et al., 2017). In addition to improved monitoring of agonist-antagonist strength ratios, it seems sensible to monitor joint ranges as a risk factor through systematic screening to identify at-risk populations early. In the present study, the inclusion of this risk factor as an inclusion criterion may have provided meaningful results in the study population, as altered scapular kinematics has also been shown to be a potential risk factor (Su et al., 2004). In contrast, there is no consensus on which of the therapeutic approaches developed to date to control the agonist-antagonist ratio is the most appropriate (Yoma et al., 2022).

The present study did not obtain the expected results in terms of muscle strength and shoulder ROM. Our results suggest that could be a positive trend in shoulder external rotation strength or range of motion. A previous study (Batalha et al., 2015) obtained significant results in terms of muscle strength. With a similar training protocol using elastic bands, they obtained significant differences in the strength of the external rotators (not the internal rotators) and in the RE/RI ratio. There are several reasons for the difference between the two studies. On the one hand, the follow-up was longer (16 vs. 8 weeks) and closer (supervised training vs. autonomous training). On the other hand, the age of the swimmers (14–15 years vs. 18–33 years) and the strength measurement was isokinetic vs. isometric.

In accordance with the above, the follow-up time seems to be the key that distinguishes the studies that obtained significant differences between groups in terms of muscle strength. Protocols lasting more than 12 weeks (Batalha et al., 2015) proved to be effective, while those with follow-ups of less than 8 weeks were unable to demonstrate differences between the proposed approaches (Hibberd et al., 2012).

However, a new unknown factor is being analysed here. It seems that adolescent swimmers (<18 years) are able to correct muscular decompensation in the shoulder with training protocols of longer duration (12–16 weeks) based on strength exercises with elastic bands (Batalha et al., 2015; Manske et al., 2015). Nevertheless, the asymmetry between the present study and these two previous studies does not allow conclusions to be drawn regarding age, but it seems important to clarify in the future whether the preventive approach could be carried out at any age or whether it would be essential to carry it out in the early stages of sporting development. On the other hand, the therapeutic approaches differ between studies, but all include resistance exercises, for external rotation of the shoulder. It appears that open kinetic chain dry training was the most appropriate for the correct development of muscle strength (Hibberd et al., 2012), so the protocol proposed in this article has the potential to be effective in overcoming the limitations found.

There are several causes that may have led to the non-significant results found. The small sample size (22 participants), in addition to a short follow-up (8 weeks), with a treatment protocol that was too simplified (1 single exercise) and supervision limited to explanation and correction on the first day of treatment. On the other hand, the variables measured could have been improved with isokinetic measurement of muscle strength.

The challenge of creating a research protocol that, while retaining simplicity to encourage swimmer accessibility and adherence, was capable of demonstrating efficacy in the control of risk factors linked to swimming for the development of shoulder pathology. Further research is still needed to create an followed protocol for balancing muscle strength and shoulder joint range in swimmers. This protocol should be available to all swimmers, regardless of competitive level, age, financial or technical means.

## 5 Conclusion

The strengthening exercise program showed minimal improvement in shoulder rotation strength and range of motion. These findings suggest that the prescribed shoulder-strengthening exercise could be a quick-beneficial dry-land training option to improve external rotation shoulder strength or range of motion, but more studies with larger sample sizes and more weeks of treatment are needed to determine the efficacy of this protocol.

## Data Availability

The original contributions presented in the study are included in the article/Supplementary Material, further inquiries can be directed to the corresponding author.

## References

[B1] Asker M. Brooke H. L. Waldén M. Tranaeus U. Johansson F. Skillgate E. (2018). Risk factors for, and prevention of, shoulder injuries in overhead sports: a systematic review with best-evidence synthesis. Br. J. sports Med. 52 (20), 1312–1319. 10.1136/bjsports-2017-098254 29581141

[B2] Bak K. (2010). The practical management of swimmer's painful shoulder: etiology, diagnosis, and treatment. Clin. J. sport Med. 20 (5), 386–390. 10.1097/JSM.0b013e3181f205fa 20818199

[B3] Batalha N. Dias S. Marinho D. A. Parraca J. A. (2018). The effectiveness of land and water based resistance training on shoulder rotator cuff strength and balance of youth swimmers. J. Hum. Kinet. 62, 91–102. 10.1515/hukin-2017-0161 29922381 PMC6006528

[B4] Batalha N. Raimundo A. Tomas-Carus P. Paulo J. Simão R. Silva A. J. (2015). Does a land-based compensatory strength-training programme influences the rotator cuff balance of young competitive swimmers? Eur. J. sport Sci. 15 (8), 764–772. 10.1080/17461391.2015.1051132 26332051

[B5] Cejudo A. Sánchez-Castillo S. Sainz de Baranda P. Gámez J. C. Santonja-Medina F. (2019). Low range of shoulders horizontal abduction predisposes for shoulder pain in competitive young swimmers. Front. Psychol. 10, 478. 10.3389/fpsyg.2019.00478 30894833 PMC6414446

[B6] Cools A. M. Vanderstukken F. Vereecken F. Duprez M. Heyman K. Goethals N. (2016). Eccentric and isometric shoulder rotator cuff strength testing using a hand-held dynamometer: reference values for overhead athletes. Knee Surg. sports traumatology, Arthrosc. 24 (12), 3838–3847. 10.1007/s00167-015-3755-9 26294055

[B7] Costa G. Silva E. Silveira A. Novaes J. Masi F. d. Conceição M. (2014). Acute effects of static and proprioceptive neuromuscular facilitation stretching on sprint performance in male swimmers. Med. dello sport 67 (1), 119–128. Avaliable at: http://www.fmsi.it/dmdocuments/MeddelloSport12014.pdf .

[B8] da Costa B. R. Armijo-Olivo S. Gadotti I. Warren S. Reid D. C. Magee D. J. (2010). Reliability of scapular positioning measurement procedure using the palpation meter (PALM). Physiotherapy 96 (1), 59–67. 10.1016/j.physio.2009.06.007 20113764

[B9] Drigny J. Gauthier A. Reboursière E. Guermont H. Gremeaux V. Edouard P. (2020). Shoulder muscle imbalance as a risk for shoulder injury in elite adolescent swimmers: a prospective study. J. Hum. Kinet. 75, 103–113. 10.2478/hukin-2020-0041 33312299 PMC7706667

[B10] Escamilla R. F. Yamashiro K. Paulos L. Andrews J. R. (2009). Shoulder muscle activity and function in common shoulder rehabilitation exercises. Sports Med. Auckl. N.Z.) 39 (8), 663–685. 10.2165/00007256-200939080-00004 19769415

[B11] Feijen S. Tate A. Kuppens K. Claes A. Struyf F. (2020). Swim-training volume and shoulder pain across the life span of the competitive swimmer: a systematic review. J. Athl. Train. 55 (1), 32–41. 10.4085/1062-6050-439-18 31935141 PMC6961642

[B12] Gaunt T. Maffulli N. (2012). Soothing suffering swimmers: a systematic review of the epidemiology, diagnosis, treatment and rehabilitation of musculoskeletal injuries in competitive swimmers. Br. Med. Bull. 103 (1), 45–88. 10.1093/bmb/ldr039 21893484

[B13] Habechian F. A. P. Van Malderen K. Camargo P. R. Cools A. M. (2018). Changes in shoulder girdle strength in 3 consecutive years in elite adolescent swimmers: a longitudinal cohort study. Braz. J. Phys. Ther. 22 (3), 238–247. 10.1016/j.bjpt.2018.01.001 29456193 PMC5993940

[B14] Hébert L. J. Maltais D. B. Lepage C. Saulnier J. Crête M. Perron M. (2011). Isometric muscle strength in youth assessed by hand-held dynamometry: a feasibility, reliability, and validity study. Pediatr. Phys. Ther. 23 (3), 289–299. 10.1097/PEP.0b013e318227ccff 21829128

[B15] Hibberd E. E. Oyama S. Spang J. T. Prentice W. Myers J. B. (2012). Effect of a 6-week strengthening program on shoulder and scapular-stabilizer strength and scapular kinematics in division I collegiate swimmers. J. sport rehabilitation 21 (3), 253–265. 10.1123/jsr.21.3.253 22387875

[B16] Hill L. Collins M. Posthumus M. (2015). Risk factors for shoulder pain and injury in swimmers: a critical systematic review. Physician Sportsmed. 43 (4), 412–420. 10.1080/00913847.2015.1077097 26366502

[B17] Holt K. Boettcher C. Halaki M. Ginn K. A. (2017). Humeral torsion and shoulder rotation range of motion parameters in elite swimmers. J. Sci. Med. sport 20 (5), 469–474. 10.1016/j.jsams.2016.10.002 27840035

[B18] Jürimäe J. Haljaste K. Cicchella A. Lätt E. Purge P. Leppik A. (2007). Analysis of swimming performance from physical, physiological, and biomechanical parameters in young swimmers. Pediatr. Exerc. Sci. 19 (1), 70–81. 10.1123/pes.19.1.70 17554159

[B19] Kluemper M. Uhl T. Hazelrigg H. (2006). Effect of stretching and strengthening shoulder muscles on forward shoulder posture in competitive swimmers. J. Sport Rehabilitation 15 (1), 58–70. 10.1123/jsr.15.1.58

[B20] Manske R. C. Lewis S. Wolff S. Smith B. (2015). Effects of a dry-land strengthening program in competitive adolescent swimmers. Int. J. sports Phys. Ther. 10 (6), 858–867. 26618065 PMC4637920

[B21] Marek S. M. Cramer J. T. Fincher A. L. Massey L. L. Dangelmaier S. M. Purkayastha S. (2014). Acute effects of static and proprioceptive neuromuscular facilitation stretching on sprint performance in male swimmers. Med. dello Sport 67 (1), 119–128.

[B22] Moeller C. R. Bliven K. C. Valier A. R. (2014). Scapular muscle-activation ratios in patients with shoulder injuries during functional shoulder exercises. J. Athl. Train. 49 (3), 345–355. 10.4085/1062-6050-49.3.10 24840585 PMC4080594

[B23] Page P. A. Lamberth J. Abadie B. Boling R. Collins R. Linton R. (1993). Posterior rotator cuff strengthening using theraband(r) in a functional diagonal pattern in collegiate baseball pitchers. J. Athl. Train. 28 (4), 346–354. 16558251 PMC1317739

[B24] Park S. Y. Park D. J. (2019). Comparison of muscular activities between subjects with and without scapular downward rotation impairment during diagonal pattern of exercises. J. Bodyw. Mov. Ther. 23 (1), 59–64. 10.1016/j.jbmt.2018.01.006 30691763

[B25] Richards J. A. Dawson T. A. (2009). Optimizing exercise outcomes: the efficacy of resistance training using conventional vs. novel movement arcs. J. strength Cond. Res. 23 (7), 2015–2024. 10.1519/JSC.0b013e3181b43aa6 19855325

[B26] Sein M. L. Walton J. Linklater J. Appleyard R. Kirkbride B. Kuah D. (2010). Shoulder pain in elite swimmers: primarily due to swim-volume-induced supraspinatus tendinopathy. Br. J. sports Med. 44 (2), 105–113. 10.1136/bjsm.2008.047282 18463295

[B27] Su K. P. Johnson M. P. Gracely E. J. Karduna A. R. (2004). Scapular rotation in swimmers with and without impingement syndrome: practice effects. Med. Sci. sports Exerc. 36 (7), 1117–1123. 10.1249/01.mss.0000131955.55786.1a 15235314

[B28] Swanik K. A. Lephart S. M. Swanik C. B. Lephart S. P. Stone D. A. Fu F. H. (2002). The effects of shoulder plyometric training on proprioception and selected muscle performance characteristics. J. shoulder Elb. Surg. 11 (6), 579–586. 10.1067/mse.2002.127303 12469083

[B29] Tessaro M. Granzotto G. Poser A. Plebani G. Rossi A. (2017). Shoulder pain in competitive teenage swimmers and it's prevention: a retrospective epidemiological cross sectional study of prevalence. Int. J. sports Phys. Ther. 12 (5), 798–811. 10.26603/ijspt20170798 29181257 PMC5685406

[B30] Tooth C. Gofflot A. Schwartz C. Croisier J. L. Beaudart C. Bruyère O. (2020). Risk factors of overuse shoulder injuries in overhead athletes: a systematic review. Sports health 12 (5), 478–487. 10.1177/1941738120931764 32758080 PMC7485028

[B31] Trinidad A. González-Garcia H. López-Valenciano A. (2021). An updated review of the epidemiology of swimming injuries. PM R J. Inj. Funct. rehabilitation 13 (9), 1005–1020. 10.1002/pmrj.12503 33010194

[B32] Walker H. Gabbe B. Wajswelner H. Blanch P. Bennell K. (2012). Shoulder pain in swimmers: a 12-month prospective cohort study of incidence and risk factors. Phys. Ther. sport. 13 (4), 243–249. 10.1016/j.ptsp.2012.01.001 23068900

[B33] Wanivenhaus F. Fox A. J. Chaudhury S. Rodeo S. A. (2012). Epidemiology of injuries and prevention strategies in competitive swimmers. Sports health 4 (3), 246–251. 10.1177/1941738112442132 23016094 PMC3435931

[B34] Weldon E. J. Richardson A. B. (2001). Upper extremity overuse injuries in swimming. A discussion of swimmer's shoulder. Clin. sports Med. 20 (3), 423–438. 10.1016/s0278-5919(05)70260-x 11494832

[B35] Witt D. Talbott N. Kotowski S. (2011). Electromyographic activity of scapular muscles during diagonal patterns using elastic resistance and free weights. Int. J. sports Phys. Ther. 6 (4), 322–332. 22163094 PMC3230160

[B36] World Medical Association (2001). World Medical Association Declaration of Helsinki. Ethical principles for medical research involving human subjects. Bull. World Health Organ. 79 (4), 373–374. 11357217 PMC2566407

[B37] Yoma M. Herrington L. Mackenzie T. A. (2022). The effect of exercise therapy interventions on shoulder pain and musculoskeletal risk factors for shoulder pain in competitive swimmers: a scoping review. J. sport rehabilitation 31 (5), 617–628. 10.1123/jsr.2021-0403 35196648

